# Does the Application of Incisional Negative Pressure Therapy to High-Risk Wounds Prevent Surgical Site Complications? A Systematic Review

**Published:** 2013-09-20

**Authors:** Michael J. Ingargiola, Lily N. Daniali, Edward S. Lee

**Affiliations:** University of Medicine and Dentistry of New Jersey, Department of Surgery, Division of Plastic Surgery, Newark, NJ

**Keywords:** incisional wound therapy, INPWT, negative pressure wound therapy, NPWT, surgical site complications, topical negative pressure

## Abstract

**Purpose:** The application of incisional negative pressure wound therapy (INPWT) to clean, closed surgical incisions is a growing clinical practice. A systematic review was conducted to evaluate the effect of INPWT on surgical sites healing by primary intention. The primary outcomes of interest are incidence of complications (infection, dehiscence, seroma, hematoma, skin necrosis, or blistering). **Methods:** Two independent reviewers performed a search of the Ovid MEDLINE and EMBASE databases from 2006 to 2012 for published articles. Supplemental searches were performed using reference lists and conference proceedings. Studies were selected for inclusion based on predetermined inclusion and exclusion criteria. Data extraction regarding study quality, demographic and clinical characteristics, and outcomes was performed independently, and data on the incidence of infection was combined using a fixed-effects meta-analysis model. **Results:** Ten (5 randomized controlled trials and 5 observational) studies were included, which investigated the outcomes of 626 incisions on 610 patients. Six studies compared INPWT with sterile dry dressings (SDDs). The literature shows a significant decrease in rates of infection when using INPWT. Results on dehiscence do show a decrease in some studies, but results are inconsistent to make a conclusion. Because of limited studies, it is difficult to make any assertions on seroma, hematoma, and skin necrosis. **Conclusions:** This systematic review shows possible evidence of a decrease in the incidence of infection with application of INPWT. Looking at other variables such as dehiscence, seroma, hematoma, and skin necrosis show no consistent data and suggest further studies in order for proper recommendations for INPWT.

Since its introduction into clinical care over a decade ago, negative pressure wound therapy (NPWT) has become a prevalent treatment modality used to promote the healing of acute wounds, chronic wounds, and skin grafts.^1^^-^3 The exact mechanism of NPWT wound healing is still an active area of research; nevertheless, evidence suggests it is accomplished through increasing blood circulation by angiogenesis, removing edema, increasing granulation tissue formation, and decreasing bacteria counts, all while increasing patient comfort.^4^^-^7 The role for the use of NPWT in the treatment of chronic wounds was further validated by a recent meta-analysis comparing NPWT to standard wound care demonstrating a significant reduction in wound size and time to healing in the NPWT group.^8^

While the use of NPWT is now an accepted therapy for secondarily healing wounds, there is an emerging body of literature describing a novel application of NPWT to surgical incisions healing by primary intention. It has been suggested that applying NPWT to a closed surgical incision (incisional NPWT [INPWT]) may hasten the healing of incision and decrease the incidence of wound healing complications, such as infection or dehiscence.^9^ Aware of the rising popularity of this novel application of the traditional NPWT, the medical supply industry has responded to this emerging market by introducing products such as the Prevena (Kinetic Concepts Inc, San Antonio, Texas) and PICO (Smith & Nephew Inc, Andover, Massachusetts) systems.

While the marketing of these products continues, the effectiveness of INPWT remains debatable. A recent Cochrane review article evaluated the efficacy of NPWT on surgical incision healing. Limited by study number and small sample size, they concluded that the effectiveness of NPWT applied to acute, primary healing surgical wounds was unclear.^10^ In their conclusion, the Cochrane reviewers made an important observation that measuring the efficacy of INPWT by the outcome of surgical incision healing is an inappropriate measurement. A more appropriate measurement is whether INPWT affects the incidence of surgical site complications.

Since the release of the Cochrane review article, several larger randomized controlled trial (RCT) studies have been published evaluating the effect of INPWT to high-risk surgical incisions.^10^ With these studies nearly doubling the population size, it is appropriate to revisit the topic. The primary clinical question asked by this systematic review is—Does application of INPWT to high-risk incisions decrease the incidence of surgical site complications, such as infection and dehiscence? We hypothesize that the use of INPWT is beneficial. To the majority of plastic surgeons, the effect of INPWT on the incidence of surgical site complications is the primary outcome of interest that will determine whether INPWT is accepted or rejected.

## MATERIALS AND METHODS

### Search strategy

Two investigators independently conducted a search encompassing the MEDLINE, EMBASE, and Cochrane databases. A primary search was performed using the following terms: negative pressure wound therapy, vacuum therapy, negative pressure dressing, topical negative pressure, negative pressure therapy, VAC, vacuum-assisted closure, vacuum pressure dressing, and vacuum-assisted closure therapy. These collected articles and then underwent a secondary search that included the following terms: primary wound closure, closed wound(s), wound healing, wound complications, flap(s), and fracture(s).

The collected articles were then filtered on the basis of article type, which were different for the 2 databases. MEDLINE articles included case reports, classical articles, clinical conferences, clinical trials, comparative studies, congresses, controlled clinical trials, evaluation studies, meta-analysis, multicenter study, RCTs, and systemic reviews. EMBASE articles included case reports, clinical articles, controlled studies, major clinical studies, clinical trials, retrospective studies, RCTs, prospective studies, case studies, systematic reviews, controlled clinical trials, and comparative studies. Additional filters included articles in the English language, from year 2006 to present, and on human subjects. Non-English language articles, articles published prior to 2006, and animal studies were excluded. Additional articles were collected for inclusion from reviewing references, abstracts, and conference proceedings.

### Selection criteria, data extraction, and quality assessment

Two nonblinded investigators (L.N.D. and M.I.) independently reviewed the studies selected from the search. Each study abstract and the full-text version was assessed for inclusion on the basis of set inclusion and exclusion criteria (Table 1). In the case of a disagreement between the 2 independent investigators, an additional independent investigator (E.S.L.) was consulted to resolve the disagreement by discussion. Cohen's Kappa Coefficient, a measure of chance-corrected agreement, was calculated to demonstrate the level of agreement between the 2 initial investigators. Interpretation was based off 0 to 0.20 as slight, 0.21 to 0.40 as fair, 0.41 to 0.60 as moderate, 0.61 to 0.80 as substantial, and 0.81 to 1 as almost perfect agreement.

The same 2 investigators performed data extraction independently using a standardized form. Again the secondary independent party was consulted to resolve any discrepancies. Data collected included study design, study subject demographic characteristics, the therapeutic intervention, and surgical outcomes of interest (Table 2).

## RESULTS

### Search results and study selection

**Figure 1:**
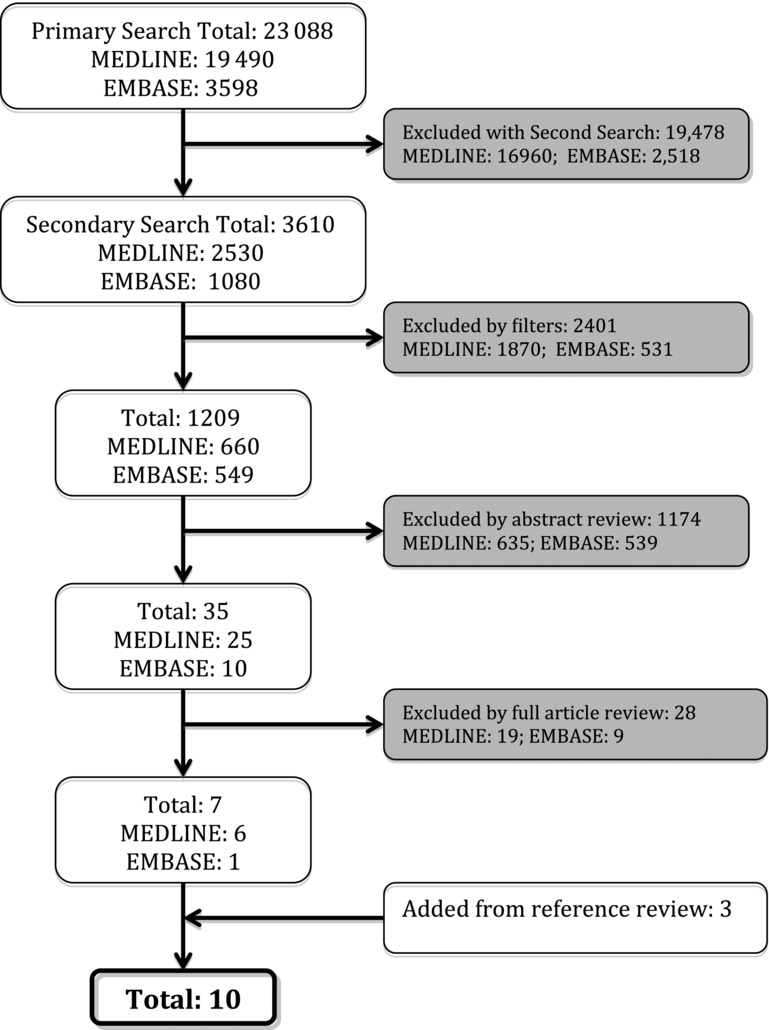
Article search and selection process.

The initial search was performed on October 1, 2012 and yielded a total of 23 088 studies. After the secondary search, application of filters, and citation review of identified articles, 10 articles remained (Fig. 1). The 2 independent investigator searches had a Cohen's Kappa Coefficient of 0.92, indicating excellent agreement.

### Study characteristics, intervention groups, and quality

Study characteristics and intervention groups of the selected studies are listed in Table 3. In total, the systematic review investigated the outcomes of 626 incisions on 610 patients. The most common study designs were as follows: RCTs (50%),^11^^-^15 retrospective observational studies (30%),^16^^-^18 and prospective observational studies (20%).^19^^,^^20^ Six of the 10 studies (60%) had a control intervention group (standard dry gauze dressing) for comparison of outcomes to the INPWT group.^11^^-^15^,^^17^ The majority of the studies evaluated the effect of INPWT to lower extremity incisions after reconstructive joint or fracture surgery (n = 7, 70%).^11^^-^15^,^^18^^,^^20^ Two studies used INPWT to sternal incisions after coronary bypass grafting,^16^^,^^19^ and 1 study evaluated its use to abdominal incisions after abdominal wall reconstruction of recurrent ventral hernias.^17^ In terms of the duration of INPWT, 7 studies applied INPWT for a predetermined 2 to 5 days at negative 125 mm Hg and then removed the dressing. 11^-^13^,^^16^^,^^17^^,^^19^^,^^20^ In 2 studies, it was applied for 2 days and then removed and reapplied for a length of time depending on the amount of drainage noted in the suction canister,^14^^,^^15^ and in one study, INPWT was continued until there was no fluid suctioned into the canister for 12 consecutive hours (typically in place for 1-3 days).^18^ Mean follow-up ranged from 1 to 21 months.

### Demographic and clinical characteristics

Eight studies provided data regarding the sex ratio of the study population,^12^^,^^14^^-^20 and 9 studies reported mean age ranging from 40 to 70 years.^12^^-^20 Body mass index, comorbid conditions such as diabetes, coronary artery disease, peripheral vascular disease, immunocompromised status, and chronic obstructive pulmonary disease were reported with variability. Nearly all studies, except one,^20^ selected study populations whose surgical incisions were considered by the investigators to be at high risk for complication because of either a large burden of comorbidities and/or by the nature of the injury, location, and known propensity for complication. All demographic and clinical characteristic data for each study are reported in Table 3.

### Outcomes and complications

Outcomes and complications are reported in Table 4, with number and percentage rate listed by study group. Outcomes and complication rates were not available for all studies.

#### Infection

The incidence of surgical site infection was the most commonly reported complication, reported by 9 studies. Four of the 5 observational studies included in the review documented a 0% incidence of infection in their study population using INPWT.^16^^,^^18^^-^20 None of these studies had control groups for comparison. Condé-Green et al^17^ demonstrated no significant difference in the rate of infection between the INPWT (1 infection, 4.3%) and the SDD (2 infections, 6%) groups. Of the 5 RCTs, 4 reported rates of infection.^17^^-^19^,^^21^ Of note, the largest study by Stannard et al^15^ showed a significant decrease in infection rate between SDD and INPWT, 18.9% and 9.9%, respectably (*P* = .049).

#### Dehiscence

Dehiscence of the surgical incision was the second most commonly reported complication (70% of studies). The rate of dehiscence ranged from 8.6% to 36.4% in the INPWT groups versus 16% to 39% in the SDD groups. Among the 4 studies with treatment and control groups that reported the incidence of dehiscence, 2 studies found a statistically significant difference.^16^^,^^19^ Condé-Green et al demonstrated an 8.7% dehiscence rate with INPWT compared to 39% with SDD (OR = 6.83, 95% confidence interval: 1.3-34.1, *P* = .014),^17^ and Stannard et al^15^ demonstrated an 8.6% percent dehiscence rate with INPWT versus 16.5% with SDD (RR = 1, 95% confidence interval: 1.03-3.55, *P* = .044).

#### Seroma and hematoma

Two studies reported data regarding the incidence of seroma.^16^^,^^21^ Pachowsky et al^13^ found a significant reduction in the incidence and mean size of seroma with INPWT (44%, 1.97 mL) versus SDD (90%, 5.08 mL) to total hip arthroplasty incisions on obese patients (*P* = .02). Stannard et al reported the incidence of mild to marked drainage from the surgical incision. While it was not specified whether this drainage represented seroma or hematoma fluid, they found a significant reduction in the number of days with greater than mild drainage from incisions with INPWT versus SDD (1.8 vs 4.8 days, respectively; *P* = .02).^14^ Condé-Green et al^17^ were the sole authors to report the incidence of hematoma. They reported a zero occurrence of hematoma in each group.

#### Skin necrosis/blistering

The incidence of skin necrosis and skin blistering was reported by 1 study. Condé-Green et al^17^ demonstrated no significant difference in skin necrosis between the INPWT and SDD groups. Howell et al^11^ experienced an increase rate of skin blistering with application of INPWT (63%) versus with SDD (12%). Blisters were described as linear at the junction between the sponge and adhesive tape most likely due to friction. The authors mentioned no specific type of therapy or prevention of these blisters.

#### Reoperation

Four studies provided data regarding the rate of reoperation. In 3 of the studies, there was a zero incidence of reoperation with the application of INPWT.^13^^,^^20^^,^^21^ Masden et al demonstrated no significant difference in the rate of reoperation between the INPWT and SDD groups (21% vs 22%, *P* = .89).^12^

#### Time to dry wound

Five studies reported data regarding the time to attainment of a dry wound. Colli and Camara^19^ found an average time of 5 days to a dry sternal incision after coronary artery bypass graft (CABG) with application of INPWT. Four of the 5 RCTs found no significant difference in the average time until attainment of a dry incision.^11^^,^^12^^,^^15^^,^^19^ A significant reduction in the time to attainment of a dry incision was demonstrated in the 2006 RCT by Stannard et al^14^ with application of INPWT to high-risk lower extremity fractures after open reduction internal fixation (1.6 vs 3.1 days, *P* = .03).

## DISCUSSION

While the use of NPWT for the treatment of open wounds healing by secondary intention and skin grafts is widely accepted and supported by the literature, application of INPWT is becoming a more ubiquitous practice of previously unclear benefit. There are several theories regarding the possible mechanism of NPWT wound healing augmentation: decreased edema, induction of mechanical stress on cells promoting cell growth, and increased microvascular blood flow.^4^^-^7 Of particular relevance, Timmers et al^21^ demonstrated more than 5-fold increase in cutaneous blood flow with application of NPWT to intact skin with pressures up to 300 mm Hg.

In 2012, the Cochrane Review conducted a systematic review of NPWT to surgical incisions using wound healing as the primary outcome of interest.^11^ Unfortunately, assessing the efficacy of INPWT by attempting to determine when a surgical incision is “completely healed” is a difficult endpoint to measure. Thus, we asked a more clinically relevant question: how does the application of INPWT affect the rate of surgical site complications?

Of all the surgical site complications measured, the incidence of infection was reported by 9 of 10 studies. None of the studies demonstrated an increased rate of infection with INPWT. Stannard et al composes a significant portion of the pooled study population for this systematic review. Stannard et al investigated the use of INPWT to high-risk lower extremity fractures, tibial plateau, pilon, and calcaneus fractures, with an established propensity for surgical site complications. Despite being a nonblinded study, Stannard et al^15^ provided level 1 evidence of a significant reduction in both the rate of infection and dehiscence with the application of INPWT in comparison to the SDD.

The effect of INPWT on the rate of dehiscence is not as thoroughly studied as the incidence of infection. No study demonstrated a significant increase in dehiscence with the application of INPWT. Of note, the largest studies with control groups for comparison, the RCT by Stannard et al^15^ and the observational study by Condé-Green et al,^17^ both demonstrated a statistically significant reduction in the rate of dehiscence with INPWT. While there is growing evidence that INPWT reduces the incidence of dehiscence, further investigation is necessary before a definitive assessment can be made.

It is difficult to make assertions and recommendations regarding the affect of INPWT on the rate of seroma, hematoma, skin breakdown, reoperation, and time until attainment of a dry wound. Despite demonstration of significant reduction in incidence and size of seroma by Pachowsky et al^13^ and in the days of mild to marked incisional drainage and time to dry wound by Stannard et al^14^ in general, these outcomes of interest were reported too inconsistently by the included studies in this review to justify any strong assertion of effect.

Howell et al^11^ were the only included study that reported an increased associated skin blistering with INPWT when applied to the incisions of total knee arthroplasty patients. The authors terminated the study early because of this high incidence of skin blistering and for concern that the blisters would increase the incidence of infection in the study patients. Despite the skin blistering, an increased rate of infection was not demonstrated. Possible explanations of the unique and high incidence of skin blistering in this study may include increased edema with total knee arthroplasty and/or a technical variation in the application of the INPWT occlusive tape. If the occlusive tape was placed across skin with tension, the superficial layers of epidermis may have been more prone to blister formation.

In regard to the anatomic area and type of surgery, the majority of the studies in this review evaluated the use of INPWT to lower extremity reconstructions. The lower extremity reconstruction studies represented the highest level of evidence with 5 RCTs. The largest proportion of these patients underwent open reduction internal fixation for reconstruction of fractures secondary to trauma. There is also an established experience of application of INPWT to arthroplasty incisions. The use of INPWT over fasciocutaneous ankle flaps is a novel use reported solely by Goldstein et al.^20^ Because of the small population size, the use of INPWT on lower extremity fasciocutaneous flaps would benefit from further study. Two observational studies investigated the use of INPWT to sternal incisions after CABG.^16^^,^^19^ Neither study reported any incidence of adverse surgical site complication nor did either have a control group for comparison. Primarily limited by the small sample size, no specific recommendations can be made regarding the application of INPWT to prevent surgical site complications post-CABG. Finally, use of INPWT to abdominal wall reconstructions is also an emerging application introduced by Condé-Green et al.^17^ The study suggests that use of INPWT to abdominal wall reconstructions may decrease the rate of dehiscence and complications overall. Nevertheless, the literature for use of INPWT on abdominal wall reconstructions would be strengthened by a RCT study with a larger population.

There is scant discussion regarding the cost of INPWT in the literature. Stannard et al^14^ estimated that the application of INPWT costs less than $500 for the mean 2.5 days of therapy per study patient. They concluded that INPWT is a cost-effective intervention as cost savings from a shortened hospital stay and prevention of postoperative surgical site infection offset the initial intervention cost. Using the data from Stannard et al, an industry study generated a theoretical health economic model estimating a potential cost savings of $5338 and $1586 per prevention of each infection and dehiscence, respectively.^22^ While these estimates may eventually be accurate in suggesting cost-efficacy of application of INPWT to high-risk surgical sites, both the Stannard and Mullins studies are theoretical estimations of cost savings. No study has yet definitively quantified and compared cost between patients receiving INPWT and SDDs.

A carefully conducted and documented literature search was performed with the goal that our methods would be transparent, reproducible, and with minimum bias. Nevertheless, we cannot rule out that there may be trials outside of our search strategy that were not included. While the 2 reviewers performed the searches and data extraction independently, they were not blinded to the studies. Finally, as only English-language papers were included for full-text review, a language bias may exist.

This review highlighted several areas where future research is necessary. In particular, further investigation into the cost associated with INPWT and its cost-efficacy is needed. Also, it remains unclear by what mechanism INPWT is conferring improved surgical site outcomes. Is it the pressure of the dressing on the skin increasing cutaneous blood flow, the semisterile occlusive environment of the dressing placed in the operating room, or is it reduction of edema decreasing tension off the healing incision edges? What type of wounds that are closing by primary intention would benefit from this therapy needs to be further evaluated in terms of complications. Is the therapy best indicated for wounds more prone to complications or patients with comorbidities? Also, although not every study specifically stated the type of NPWT equipment used it would be important to look at a standard to compare results or compare different types of machines available and if complication results vary. Finally, there are established and validated models estimating the risk of surgical site complication in certain surgical populations, that is, the Fowler score to estimate the risk of sternal infection after cardiac surgery.^23^ Ideally, future studies may consider incorporating such a model to demonstrate modification of the rate of infection with use of INPWT.

## CONCLUSIONS

Many studies are emerging attempting to show decreased rates of complications in patients using INPWT. Currently, as the present body of literature focuses on patient cohorts with significant comorbidities and risk factors for surgical site complication, there is insufficient evidence to support the generalized application of INPWT to all surgical incisions. However, this review suggests the use of INPWT is a safe, well-tolerated intervention that may offer clinical benefit.

## Figures and Tables

**Table 1:** Inclusion and exclusion criteria

Inclusion criteria	Exclusion criteria
1. Application of NPWT to surgical incisions after incision closure in the operating room; Incisions healing by primary intention.	1. Application of NPWT to open wounds or skin grafts healing by secondary intention.
2. Study population of ≥10	2. Study population <10
3. English language article from 2006 to date of search	3. Non-English language articles and articles published prior to 2006
4. Human subjects	4. Animal studies

**Table 2:** Data items extracted

Study design
Patient demographics
Type of surgery and location of surgical incision
Intervention
Length of NPWT or control dressing application
Dressing configuration
NPWT pressure setting
Criteria to stop NPWT and control dressing
Outcomes
Mean follow-up time
Infection
Dehiscence
Seroma and hematoma
Skin breakdown (necrosis, blister)
Reoperation
Time to dry wound

**Table 3:** Included studies, methodology, and demographics*

Reference	Study design	No. of incisions; No. of patients	Area of surgical incision; Type of surgery	Duration of application and pressure, mm Hg	Mean follow-up, mo	Postop incision intervention group(s) (No. of incisions)	
Stannard et al^14^	RCT	44; 44	LE; ORIF for LE trauma reconstruction	2 d + DDD; NR	NR	SDD (n = 24) INPWT (n = 20)	
Reddix et al^18^	R	19; 19	LE; ORIF acetabular fracture	VDD; -75	21	NAG + INPWT (n = 19)	
Atkins et al^16^	R	57; 57	S; CABG	4 d; -125	NR	NAG + Silver foam INPWT (n = 57)	
Goldstein et al^20^	PC	17; 10	LE; Fasciocutaneous flaps	4 d; -125	2.9	INPWT (n = 17)	
Pachowsky et al^13^	RCT	19; 19	LE; THA	5 d; -125	NR	SDD (n = 10) INPWT† (n = 9)	
Colli and Camara^19^	PC	10; 10	S; CABG	5 d; -125	1	INPWT† (n = 10)	
Howell et al^11^	RCT	60; 51	LE; TKA	2 d; -125	12	SDD (n = 36) INPWT (n = 24)	
Masden et al^12^	RCT	81; 81	A, B, LE; Closure of vascular bypass wounds and LE amputations	3 d; -125	3.8	SDD (n = 37) NAG + INPWT (n = 44)	
Stannard et al^15^	RCT	263; 263	LE; ORIF for LE trauma reconstruction	2 d + DDD; -125	NR	SDD (n = 122)	
						INPWT (n = 141)	
Conde-Green et al^17^	R	56; 56	A; Abdominal wall reconstruction	5 d; -125	15	SDD (n = 33) NAG + INPWT (n = 23)	
	Demographic and clinical characteristics						
Reference	Age range, y	Gender (male; female), %	Mean BMI	DM, %	PVD; CAD, %	Immunocom-promised, %	Smoking; COPD, %
Stannard et al^14^	19-78 (mean, 41)	73; 27	NR	NR	NR	NR	NR
			NR	NR	NR	NR	NR
Reddix et al^18^	20-72 (mean, 41)	21; 79	48.7	NR	NR	NR	NR
Atkins et al^16^	NR (mean, 60)	89.5; 10.5	35.3	54.4	21; 100	NR	NR
Goldstein et al^20^	40-83 (mean, 59)	60; 40	NR	40	NR	NR	NR
Pachowsky et al^13^	NR (mean, 71)	NR	NR	NR	NR; NR	NR	NR
Colli and Camara^19^	NR (mean, 66)	NR	NR	NR	NR; NR	NR	NR
Howell et al^11^	55-73 (mean, 66)	50; 50	50% with BMI 30-40 Kg/m^2^	NR	90; 100	NR	NR; 30
	NR	NR	100% with BMI >30 Kg/m^2^	0	NR	NR	NR
Masden et al^12^	38-86 (mean, 61)‡	62;38‡	32.1‡	64.9‡	54‡; NR	8.1‡	13.5‡; NR
	40-101 (mean, 61)‡	70;30‡	31‡	79.6‡	47.7‡; NR	9.1‡	18.2‡; NR
Stannard et al^15^	18-80 (mean, 43)‡	65; 35‡	NR	3.3	NR; NR	NR	52‡
			NR	7.8	NR; NR	NR	
Conde-Green et al^17^	3-81 (mean, 55)	41; 59‡	36.1‡	21‡	NR; 10.8‡	3.1‡	13.8‡; 5.6‡
	21-72 (mean, 54)		36.4‡				

*A indicates abdomen; B, back; BMI, body mass index; CABG, coronary artery bypass graft; COPD, chronic obstructive pulmonary disease; DDD, days depending on drainage; INPWT, incisional negative pressure wound therapy; LE, lower extremity; NA, not applicable; NAG, nonadhesive gauze; NR, not reported; ORIF, open reduction internal fixation; PC, prospective cohort; R, retrospective; RCT, randomized controlled trial; S, sternum; SDD, sterile dry dressing; THA, total hip arthroplasty; TKA, total knee arthroplasty.

†Prevena incisional negative pressure wound therapy system (Kinetic Concepts, Inc, San Antonio, Texas)

‡No significant difference between groups (individual subgroup data not reported).

**Table 4:** Outcome measures and complications*

Reference	Postoperative incision intervention group(s) (No. of incisions)	Infection, %	Dehiscence, %	Seroma; hematoma, %	Skin necrosis; Skin blistering, %	Reoperation, %	Time to dry wound, d
Stannard et al^14^	SDD (n = 24)	3 (12.5)	4 (16.7)	4.8 d‡	NR; NR	NR	3.1
	INPWT (n = 20)	3 (15)	4 (20)	1.8 d‡ (*P* = .02)	NR; NR	NR	1.6 (*P* = .03)
Reddix et al^18^	NAG + INPWT (n = 19)	0	0	NR; NR	NR; NR	NR	NR
Atkins et al^16^	NAG + Silver foam INPWT (n = 57)	0	NR	NR; NR	NR; NR	0	NR
Goldstein et al^20^	INPWT (n = 17)	0	2 (11.8)	NR; NR	NR; NR	NR	NR
Pachowsky et al^13^	SDD (n = 10)	NR	NR	9 (90); NR	NR; NR	NR	NR
	INPWT† (n = 9)	NR	NR	4 (44) (*P* = .021); NR	NR; NR	NR	NR
Colli and Camara^19^	INPWT† (n = 10)	0	0	NR; NR	NR; NR	0	5
Howell et al^11^	SDD (n = 36)	1 (2.8)	NR	NR; NR	NR; 3 (12)	0	4.1
	INPWT (n = 24)	1 (4.2)	NR	NR; NR	NR; 15 (63)	0	4.3
Masden et al^12^	SDD (n = 37)	5 (13.5)	11 (29.7)	NR; NR	NR; NR	8 (22)	4.1
	NAG + INPWT (n = 44)	3 (6.8)	16 (36.4)	NR; NR	NR; NR	9 (21)	4.3
Stannard et al^15^	SDD (n = 122)	23 (18.9)	20 (16.5)	NR; NR	NR; NR	NR	3
	INPWT (n = 141)	14 (9.9) (*P* = .049)	12 (8.6) (*P* = .044)	NR; NR	NR; NR	NR	2.5
	SDD (n = 33)	2 (6)	13 (39)	4 (12); 0	6 (18); NR	NR	NR
Conde-Green et al^17^	NAG + INPWT (n = 23)	1 (4.3)	2 (8.7) (*P* = .014)	0; 0	2 (8.7); NR	NR	NR

*Abbreviations are explained in the first footnote to Table 3.

†Prevena incisional negative pressure wound therapy system (Kinetic Concepts Inc, San Antonio, Texas).

‡Days of drainage from incision greater than minimal (>2 quarter-sized drops of drainage).
